# Supplementary figures and images for: Predictive Value of Modified Glasgow Prognostic Score and Persistent Inflammation among Patients with Non-Small Cell Lung Cancer Treated with Durvalumab Consolidation after Chemoradiotherapy: A Multicenter Retrospective Study
Source: Cancers (Basel). 2023 Sep 1;15(17):4358. doi: 10.3390/cancers15174358 (PMC10486354; doi:10.3390/cancers15174358)

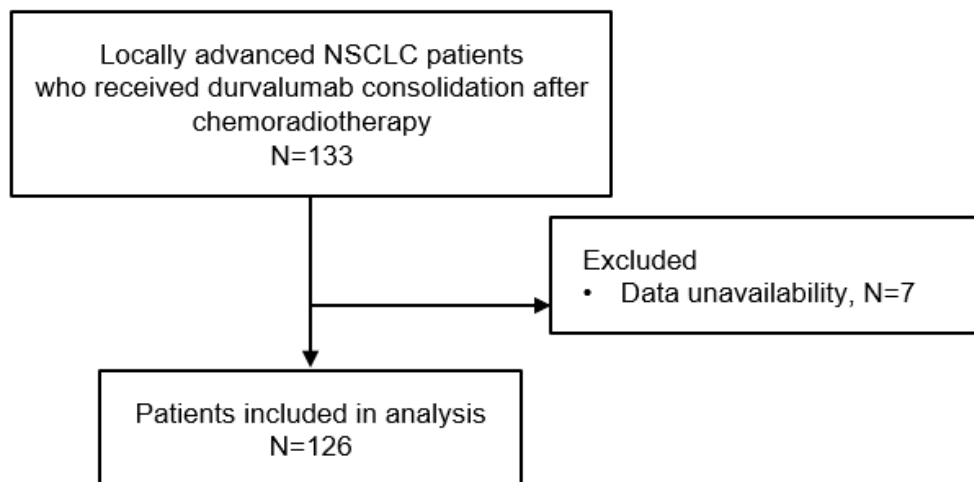

**Figure S1.** Patient flow-diagram.

Supplement: Supplementary file 1 [file cancers-15-04358-s001.zip › cancers-2511328-supplementary.pdf]
